# COVID-19 highlights the need for universal adoption of standards of medical care for physicians in nursing homes in Europe

**DOI:** 10.1007/s41999-020-00347-6

**Published:** 2020-06-17

**Authors:** Desmond O’Neill, Robert Briggs, Iva Holmerová, Olafur Samuelsson, Adam L. Gordon, Finbarr C. Martin, The Special Interest Group in Long Term Care of the European Geriatric Medicine Society

**Affiliations:** 1 Centre for Ageing, Neuroscience and the Humanities, Trinity College Dublin, Trinity Centre for Health Sciences, Tallaght University Hospital, Dublin, D24 NR0A Ireland; 2 Trinity College Dublin, Dublin, Ireland; 3 Charles University, Prague, Czech Republic; 4 Landspitali University Hospital, Reykjavik, Iceland; 5 Division of Medical Sciences and Graduate Entry Medicine, University of Nottingham, Nottingham, UK; 6 Population Health Sciences, King’s College London, London, UK

**Keywords:** MeSH, Nursing home, Standard of care, Physicians, COVID-19

## Abstract

The nursing home sector has seen a disproportionately high number of deaths as part of the COVID-19 pandemic. This reflects, in part, the frailty and vulnerability of older people living in care homes but has also, in part, been a consequence of the failure to include care homes in the systematic planning of a response to COVID, as well as a measure of neglect of standards and quality improvement in the sector. In response, the EUGMS published a set of medical standards of care developed in consultation with experts across its member national societies in 2015. The standards consisted of seven core principles of medical care for physicians working in nursing homes as a first step in developing a programme of clinical, academic and policy engagement in improving medical care for older people who are living and frequently also dying as residents in nursing homes. The gravity of the concerns arising for nursing home care from the COVID-19 pandemic, as well as emerging insights on care improvement in nursing homes indicate that an update of these medical standards is timely. This was performed by the writing group from the original 2015 guidelines and is intended as an interim measure pending a more formal review incorporating a systematic review of emerging literature and a Delphi process.

## Key summary points

**Aim:**

The gravity of concerns arising for nursing home care from the COVID-19 pandemic mandates urgent review of medical standards for nursing homes.

**Findings:**

Physicians providing medical care to nursing home residents should have a formal competence in geriatric medicine and old age psychiatry.

**Message:**

The coordination of the broad range of complexities of care in nursing homes requires clearly specified clinical leadership commensurate with the range of services needed.

The nursing home sector has seen a disproportionately high number of deaths as part of the COVID-19 pandemic. This reflects, in part, the frailty and vulnerability of older people living in care homes but has also, in part, been a consequence of the failure to include care homes in the systematic planning of a response to COVID. This has compounded longstanding issues with funding, staffing, and access to expertise in geriatric medicine and gerontological nursing in long-term care [1]. With the pandemic, there is the additional challenge of community life exposing care home residents to specific increased risks of this easily transmissible virus.[2].

Recognition that providing organised, gerontologically attuned medical care for older people requiring nursing home (NH) care is a significant challenge is not new [3]. The urgency of so doing is partly due to the increasing numbers of people requiring residential care. By 2040, the world is projected to have 1.3 billion people over 65 years old (14% of the total population) compared to 506 million in 2008 [4]. While the majority of older people spend most of their later years living healthy, independent lives, the chances of needing NH care increases significantly with age. Currently around 4% of older people in Europe live in NHs [5] or in residential care institutions The number of people requiring institutional care is projected to increase by 127% and 111% in Germany and the UK respectively between the years 2000 and 2050 [6]. The ANCIEN (Assessing Needs of Care in European Nations) research project involving 20 EU member states provided estimates of future long-term care needs of older Europeans [7]. In this, a doubling of need for nursing and residential care beds from 2010 to 2060 was predicted in the Netherlands, and a 50% increase in Poland, the latter starting from a low base as is widespread in central and eastern European countries. This is necessitated in part by demographic changes affecting numbers and proportion of population age groups but also the changing nature of the health and healthcare needs of older people.

The nature of what consists a nursing home defies easy characterization, and although a range of terminologies is used in various countries for nursing homes, including ‘care homes’: in the interests of developing a common interdisciplinary language, the term ‘nursing home’ is adopted in line with the International Association of Gerontology and Geriatrics and American Medical Directors Association position statement [8]. In addition, we are mindful that care in nursing homes in Europe is provided by a range of medical specialties, from general practitioners, through dedicated nursing home doctors, to geriatricians [9]: however, our Special Interest Group liaises with all countries in Europe, and our deliberations are formed with a view to applicability in all of these settings.

The European Ageing Report 2015 from the European Commission and the Economic Policy Committee stated that determined policy action on long-term care systems was needed in Europe [10]. It is almost a decade since the World Health Organization and the International Association of Gerontology and Geriatrics produced an important strategy document for improving care and research in nursing homes with a global perspective [3]. However, each profession engaged with nursing home healthcare needs to develop its own standards. Improving provision and standards of long-term care generally including care homes was a central pillar of the Global strategy and action plan on ageing and health of the World Health Organization (WHO) published in 2016 and adopted by member countries at the World Health Assembly of 2016 [11].

Most people in nursing homes need help with personal care in daily life, as illustrated by the SHELTER study, a 12 months prospective study of 4156 residents in 57 nursing homes in 7 EU countries (the Czech Republic, England, Finland, France, Germany, Italy, the Netherlands) and Israel, using the InterRAI Long-term Care Facility assessment tool (interRAI LTCF). Disability in activity of daily living and cognitive impairment was observed in 81.3% and 68.0% of residents, respectively. Also common were responsive behaviour previously described as behavioural symptoms (27.5% of residents], falls (18.6%), pressure ulcers (10.4%), pain (36.0%) and urinary incontinence (73.5%) [12]. The care home resident population had a higher prevalence of significant functional limitations in comparison to age-matched community dwelling peers [13]

These characteristics result in considerable clinical complexity [14]. For example, over 85% of NH residents presenting to an Irish emergency department (ED) had at least four significant medical comorbidities, as well as a pre-existing diagnosis of dementia in almost two thirds [15]. They also have high rates of delirium during acute illnesses [16], as well as high rates of frailty [17] and depression:

Despite all this, NH residents receive medical care which is less organised than their community dwelling counterparts with poorer monitoring of chronic disease and higher rates of unnecessary prescribing and especially inappropriate sedation [18]. Although Comprehensive Geriatric Assessment (CGA) is ideally the basis of providing care in nursing homes, its implementation is challenged by a range of factors [19].Whilst high quality care for individuals with these characteristics and their associated healthcare needs requires an integrated multidisciplinary approach promoted by CGA, each profession engaged with nursing home care can contribute by developing and promoting its own standards of care in harmony with the tenets of CGA.

The EuGMS, as a society representing national organisations for geriatric medicine in Europe, instituted the Special Interest Group for Long Term Care in 2011, providing a European focus for the development of standards of care, research and education for the medical care of residents of NHs. We reported from a survey across members that only 12% of EUGMS countries had written medical care standards for physicians applicable to nursing home care provided by professional organizations [20]. In response, the EUGMS published a set of medical standards of care developed in consultation with experts across its member national societies [21]. These standards comprised of seven core principles of medical care for physicians working in NHs as a first step in developing a programme of clinical, academic and policy engagement in improving medical care for older people who are living and frequently also dying as residents in nursing homes. Adoption of these standards is complicated by the fact that medical care is provided by a heterogeneous range of physician disciplines across Europe, in the main general practitioners, but also medical officers, internists or geriatricians [9]: only in the Netherlands is there a specific post-graduation career and training path to produce NH specialists (called ‘elderly care physicians’, distinct from geriatricians who have a longer training incorporating acute internal medicine of older people) [22]. Here we highlight the need for progress in widespread adoption and promotion of the EuGMS standards by drawing on the experience of the COVID pandemic.

The gravity of the concerns arising for nursing home care from the COVID-19 pandemic, as well as emerging insights on care improvement in nursing homes indicate that an update of these medical standards is timely. This was performed by the writing group for the original 2015 guidelines and is intended as an interim measure pending a more formal review incorporating systematic review of emerging literature and a Delphi process.

*1) All patients under consideration for admission to nursing home care should have an assessment by a specialist in geriatric medicine or old-age psychiatry or both if necessary, prior to admission.* This assessment aims to detect and remediate illness and functional loss so as to clarify whether NH admission can be avoided or deferred. It would also better delineate care needs for the older person, whether continuing to live in the community or entering the NH. Such assessments have been shown to reduce deterioration in physical functioning and reduce need for contact with NH and emergency services in those assessed, as well as reducing levels of distress amongst their carers [23]. The role of the assessor, and associated multi-disciplinary team as required, is to act as a gate-keeper to NH care and advocate for the older person, ensuring older people receive continuing care in an environment appropriate to their needs and wishes. While old age psychiatry is not as yet recognized as a specialty in all European countries, the psychiatry section of the European Union of Medical Specialists recommended its development in 2013, and in general in countries where it is established it is accepted that geriatricians and old age psychiatrists will sufficiently understand their scope of practice to make due referral to the other specialism as required.

*2) The coordination of the broad range of complexities of care, including liaison between primary, secondary care, public health, laboratory sciences and occupational health, as well as the need to incorporate resilience and reserve in the nursing home sector mandate the need for clearly specified clinical leadership for both individual nursing homes and for nursing homes within specified regions commensurate with provision of the range of services needed.* The COVID-19 pandemic has uncovered the complexity of coordinated liaison between healthcare staff in nursing homes, ranging from the challenges of screening staff—which requires consideration of backfill for staff quarantined when screening positive as well as effective inputs from occupational health and specialists in public health and infectious diseases—to the planning of the resilience and reserve needed for responding to pandemics. It is clear that this cannot happen effectively without a mechanism for oversight and leadership for both individual nursing homes and also nursing homes within a region commensurate with provision of the range of services needed [24]. One possible model is that of the medical director role developed in the USA following scandals over quality of care in nursing homes in the 1980′s, a mode of oversight in continuing development [25]. Such leadership needs be suitably structured, trained and supported for complex task of developing appropriate liaison, relationships, quality improvement and training within the cultures of care in nursing homes and among supporting services [26].

*3) Given the complexity of care associated with older people in nursing homes, physicians providing medical care to nursing home residents should have a formal competence in geriatric medicine and old age psychiatry. *This competence should include a core set of knowledge, skills and attitudes which prepares physicians, in the main likely to be general practitioners, for the complexity of care in later life, spanning prevention, health gain, health maintenance and palliative care. Currently only 20% of health services in the EUGMS countries have a requirement for specific training in geriatric medicine for doctors working in NHs [20]. This specific training may take the form of a defined training pathway for NH physicians as in the Netherlands [22] or else through the added qualifications such as diplomas in geriatric medicine for general practitioners as in Ireland [27]. In addition, training in core aspects of nursing home medicine should be incorporated into undergraduate medical training and higher specialist training.

*4) The medical care needs to be supported in the nursing home by nurses who have gerontological training, including training in dementia and palliative care, and care attendants who have due training in the care of older people.* Effective care of older people in care homes requires integrated working by a multidisciplinary team of experts [19]. This requires support of nursing with training and experience in care of older people (gerontological nurses). Indeed, the employment of agency staff with no background training in gerontology has been associated with lower quality of care in NHs [28]. High turnover of nursing staff is a well-recognised problem in NHs and to promote working in the NH sector as a stimulating, rewarding career path, nurses need to receive appropriate guidance and support [29].

*5) The medical care needs to be supported by associated disciplines, and in particular physiotherapy, occupational therapy, speech and language therapy [including skills in dysphagia assessment and management], clinical nutrition and pharmacy, dentistry, ophthalmology and audiology as a minimum, and access to other professions—social work and psychology—as required.* The multi-disciplinary team (MDT) has an essential role in maintenance of mobility and function [30], contracture prevention [31], seating and pressure care and nutritional support [32], prevention of aspiration [33], and polypharmacy. Full MDT support is needed to ameliorate the functional loss that often parallels acute illness in NH residents, from decompensation of gait disorders in the context of delirium to swallow deterioration and aspiration. If therapy is not available in this setting, patient care will suffer, and referrals of NH patients to acute care will increase unnecessarily. Such MDT input may be from teams shared with community services or through teams dedicated to one or more nursing homes. Building a collaborative and shared learning approach between “external MDTs” and care home staff increases the quality of health care provided [26].

*6) The medical care also needs to be supported by specialist gerontology services, including geriatricians, old age psychiatry and clinical nurse specialists as well as specialist palliative care support. *The complexity of NH residents is such that access to specialist care (beyond the competency of treating NH physician) will be required. Access to expertise in areas such as co-ordinating rehabilitation, managing multi-morbidity and behavioural disturbance, and palliative care [34], is crucial. This expertise should ideally be provided on-site as required as an adjunct to the treating physician, with the development of telemedicine services potentially providing an alternative option for access to specialist opinion [35].

This need for specialist support has been demonstrated in response to the pandemic by the rapid implementation in South West France of a COVID-19 support platform, linking the expertise of hospital geriatric departments to the teams providing care in nursing homes, enabled by an established between these hospital geriatric units and nursing homes, both public or private [36]: similar developments have occurred in a number of European countries.

*7) The process of maintaining resident medical and nursing records should be gerontologically attuned so as to reflect the needs of this patient group and support clinical decision-making. *One of the major barriers to medical and nursing home care has been the lack of systematic and comprehensive recording of the care needs of residents. Adoption of an assessment tool that is resident-centred rather than focused on gathering information for reimbursement would be useful in this regard [37]. Among the qualities desirable in such medical and nursing records are that they should be clinically useful; reasonably brief; computerised in a manner consistent with ICD-10, the International Classification of Function, and the Systematized Nomenclature of Medicine (SNOMED), the underlying code of electronic patient records; support individual care plans for common conditions; help generate dependency levels; assist regulatory authorities; and allow for the collection of meaningful data [38]. These tools have the ability to support improved standards and research in NHs, although they will not do so on their own, and need to be implemented in a context which recognises the importance of appropriate philosophies of care, staff training and resourcing.

*8) Appropriate schedules should be maintained for preventive interventions (such as vaccination), monitoring of chronic diseases, and regular clinical review and medication review. *Considering that the majority of presentations of NH residents to the ED are in the context of decompensation of a chronic disease rather than a de novo illness, regular monitoring of chronic disease is vital [39]. The concern is that this task falls between the two stools of hospital-based specialist clinics and regular general practitioner (GP) input. For example, almost 70% of NH managers identified a lack of formal follow-up procedures for NH residents with stroke disease, one of the most prevalent comorbidities in NH residents [40]. Additionally, despite being reviewed more often by their GP than community dwelling older people, NH residents are less likely to be followed up by their GP after this review [41]. A structured format for review of chronic disease such as osteoporosis, heart failure or hypothyroidism, as well as a medication review and appropriately executed and supported advance care planning, seems the way forward, although this will obviously require increased support in terms of time and finances for the physician, responsible.

These guidelines should represent the minimum standards of medical care for physicians engaging with the care of NH residents. While it is important not to over-medicalise an environment that is also a home to its residents, we must also recognise this cohort as vulnerable, with levels of disability and medical comorbidity that dictate a need for structured, organised medical care. Given the growing numbers and increasing complexity of multi-morbidity and functional loss among frail older people in Europe requiring NH care it is clear that the current lack of attention to standards and organisation of medical care in these facilities is no longer acceptable.

While it is true that long-term care policies and measures are the responsibility of the individual EU member states and that the huge differences in long-term care provision across the EU pose barriers to EU policy coordination, it is hoped that these updated guidelines represent an important step towards improving medical care in NHs, and may also prove useful in a wider European and global context.
